# Supplementary material for: Overload of the lower limbs of firefighters as a result of external conditions
Source: PLoS One. 2025 Dec 29;20(12):e0338626. doi: 10.1371/journal.pone.0338626 (PMC12747346; doi:10.1371/journal.pone.0338626)
Supplement: Attachment — Submitted filename: Response to Reviewers.docx [file pone.0338626.s001.docx]

**Response to Reviewers**

Thank you very much for your reviews and all your comments regarding our article. All of them have been taken into account. The article has been revised and expanded according to the editors' and reviewers' suggestions.

**Responses to reviewer comments**

**Reviewer #1:**

1-The age distribution of participants in the abstract and the text of the article is different and not the same.

**Answer** – It was our mistake and has been corrected.

2- I suggest that a schematic image or time line of the study protocol and the jumps be included in the text of the article.

**Answer** – Thank you for your suggestion, it has been taken into account and the research procedure diagram has been included in the manuscript.

3. There is no discussion anywhere about the shoes of the participants. Are the shoes different in athletic and firefighter uniforms?

**Answer** – Thank you very much for this comment. Of course, this wasn't clearly stated. This has been corrected and supplemented in the research method.

4. Did anyone drop out of your study? What are the exclusion criteria?

**Answer** – No one drop out from the study, but six individuals were not qualified for the main study. Exclusion criteria were included in the research procedure diagram Fig 1 in manuscript.

**Reviewer #2:**

The authors have assessed three age groups pf firefighters with no support for age groups or age effect in the introduction. I can’t understand why three age groups of firefighters are included. Please clarify.

**Answer** – Occupational demands in firefighting persist across a wide age span, from younger recruits to experienced older personnel. Age-related changes in neuromuscular control, balance, and muscular strength may alter the way external loads are managed, potentially increasing susceptibility to overload. Despite this, limited research has examined how age interacts with physical demands typical of firefighting tasks, such as impact landings under external load.

**Abstract**
The structure of abstract does not follow a standard manner. This part must be rewritten.
Background, it is not logical that you completed this part with a question. Better to provide a background regarding to firefighters and their fire protection equipment which leads to overload and then your question. Results, please provide your findings objectively with p-values.

**Answer** – The abstract was revised in accordance with the reviewer's suggestions and the journal's guidelines.

**Introduction**
The flow of idea needs to be strengthened. After reading this section for several times, I cant understand what is the novelty and the rationale. This is simple to say that the authors have investigated the effect of two levels of clothes and three age groups on GRF. Please provide support for your outcomes. Moreover, I did not find any support for knee joint stability (H:Q) through the introduction. In this case, I could think the authors have collected any available variable with no rationale. Please rewrite the introduction accordingly.

**Answer** – Thank you for your suggestion. In the introduction part, explanations and rationale of the novelty were added.

**Methods**
Oreder of conditions is not randomized. Please acknowledge it in the study limitations.

**Answer** – Yes, You're right, that the order of conditions is not randomized and it is limitations of our study. We took into account the fire stations designated by the main fire service office. Your comment has been acknowledge it in the study limitations.

L130-137 It must not be appeared here.

**Answer** – The analysis of anthropometric variables was transferred to the research results

L215 and height

**Answer** – It was added

**Discussion**
L229 is unknown

L303-308 remove

**Answer** – It has been removed

L318-326 please discuss about your results, not review the literaturę

**Answer** – Some suggested text has been removed and some has been moved.

L335-336 different types of evaluating H/Q are existing. Mostly are isokinetic assessments. Here, based on your values and existing literature, please judge whether the values are low or not.

The reference Mbada et al., 2021 is not in the references list.

The reference Tabor et al., 2021 is not valid for your claim.

L341 please add reference

L352 Vy et al is not in the references list

**Answer** – I agree that we cannot base our theories on our own beliefs and our other research. Therefore, I have supplemented these theories with research from other authors. The reference Mbada et al. 2021 is in 25th position references list. There was a typo in the name. It has been corrected. The reference Vu et al. 2021 is in 10th position references list.

L360-361 you have not plantar flexion data. Please avoid leaps

L364-367 you can’t use these sentences

L367-373 please don’t use leaps

L373-379 please discuss your results

L419 please remove it and its related phrases

**Answer** – We agree with the reviewer's suggestion. The highlighted passages have been removed from the article.

The style of referencing does not match between the text and bibliographic sections. Through the text is author, year, but, the bibliographic is numbered. Also, please ensure that all referenced are included in the bibliographic section.

**Answer** – The references list has been supplemented and corrected.
