# Supplementary material for: Overload of the lower limbs of firefighters as a result of external conditions
Source: PLoS One. 2025 Dec 29;20(12):e0338626. doi: 10.1371/journal.pone.0338626 (PMC12747346; doi:10.1371/journal.pone.0338626)
Supplement: Attachment — Submitted filename: Dear editor.docx [file pone.0338626.s002.docx]

Dear editor

PLOS One

Thank you for choosing me to review this article.

I am confused about reviewing this article. The file sent to me includes the authors' corrections. I suggest that after making the corrections, you send me the final file without asking for the originals of the previous reviewers. I was not the previous reviewer of this article.

However, my review of this study is as follows:

1- The age groups of the people selected in the main text of the article and the abstract are not the same.
2- What is the criteria for selecting people in the age category?
3- Nothing is said about the gender of the people.
4- Detailed study inclusion and exclusion criteria should be stated.
5- timeline and consort diagram of study must be present
6- Did the participants have musculoskeletal problems before the study? If so, what instrument was used and how was it measured?
